# Disruption of poly (3-hydroxyalkanoate) depolymerase gene and overexpression of three poly (3-hydroxybutyrate) biosynthetic genes improve poly (3-hydroxybutyrate) production from nitrogen rich medium by *Rhodobacter sphaeroides*

**DOI:** 10.1186/s12934-019-1088-y

**Published:** 2019-02-26

**Authors:** Jyumpei Kobayashi, Akihiko Kondo

**Affiliations:** 1 Graduate School of Science, Technology and Innovation, Kobe University, 1-1 Rokkodaicho, Nada-ku, Kobe, Hyogo 657-8501 Japan; 2 Department of Chemical Science and Engineering, Graduate School of Engineering, Kobe University, 1-1 Rokkodaicho, Nada-ku, Kobe, Hyogo 657-8501 Japan; 3 RIKEN Center for Sustainable Resource Science, 1-7-22 Suehiro-cho, Tsurumi-ku, Yokohama, Kanagawa 230-0045 Japan

**Keywords:** *Rhodobacter sphaeroides*, Poly (3-hydroxyalkanoates), Poly (3-hydroxybutyrate), Acetyl-CoA acetyltransferase, Acetoacetyl-CoA reductase, Poly (3-hydroxyalkanoate) polymerase, Poly (3-hydroxyalkanoate) depolymerase

## Abstract

**Background:**

Due to various environmental problems, biodegradable polymers such as poly (3-hydroxybutyrate) (PHB) have gained much attention in recent years. Purple non-sulfur (PNS) bacteria have various attractive characteristics useful for environmentally harmless PHB production. However, production of PHB by PNS bacteria using genetic engineering has never been reported. This study is the first report of a genetically engineered PNS bacterial strain with a high PHB production.

**Results:**

We constructed a poly (3-hydroxyalkanoate) depolymerase (*phaZ*) gene-disrupted *Rhodobacter sphaeroides* HJ strain. This *R. sphaeroides* HJΔ*phaZ* (pLP-1.2) strain showed about 2.9-fold higher volumetric PHB production than that of the parent HJ (pLP-1.2) strain after 5 days of culture. The HJΔ*phaZ* strain was further improved for PHB production by constructing strains overexpressing each of the eight genes including those newly found and annotated as PHB biosynthesis genes in the KEGG GENES Database. Among these constructed strains, all of gene products exhibited annotated enzyme activities in the recombinant strain cells, and HJΔ*phaZ* (*phaA3*), HJΔ*phaZ* (*phaB2*), and HJΔ*phaZ* (*phaC1*) showed about 1.1-, 1.1-, and 1.2-fold higher volumetric PHB production than that of the parent HJΔ*phaZ* (pLP-1.2) strain. Furthermore, we constructed a strain that simultaneously overexpresses all three *phaA3*, *phaB2*, and *phaC1* genes; this HJΔ*phaZ* (*phaA3*/*phaB2*/*phaC1*) strain showed about 1.7- to 3.9-fold higher volumetric PHB production (without ammonium sulfate; 1.88 ± 0.08 g l^−1^ and with 100 mM ammonium sulfate; 0.99 ± 0.05 g l^−1^) than those of the parent HJ (pLP-1.2) strain grown under nitrogen limited and rich conditions, respectively.

**Conclusion:**

In this study, we identified eight different genes involved in PHB biosynthesis in the genome of *R. sphaeroides* 2.4.1, and revealed that their overexpression increased PHB accumulation in an *R. sphaeroides* HJ strain. In addition, we demonstrated the effectiveness of a *phaZ* disruption for high PHB accumulation, especially under nitrogen rich conditions. Furthermore, we showed that PNS bacteria may have some unidentified genes involved in poly (3-hydroxyalkanoates) (PHA) biosynthesis. Our findings could lead to further improvement of environmentally harmless PHA production techniques using PNS bacteria.

**Electronic supplementary material:**

The online version of this article (10.1186/s12934-019-1088-y) contains supplementary material, which is available to authorized users.

## Background

Poly (3-hydroxyalkanoates) (PHA) are biodegradable polymers produced and accumulated in various kinds of microorganisms as a carbon and energy storage material. PHA have physical properties similar to those from conventional fossil fuel-based thermoplastics [1]. Therefore, PHA are more attractive as environmentally harmless polymers than conventional oil-based ones. Furthermore, because of existing problems with microplastic, a ban on oil-based and single-used plastics was recently approved in the European Union. From this background, the demand for PHA is expected to increase in the future.

A lot of studies on PHA production by various kinds of microorganisms have been reported so far [2–6]. Among these microorganisms, purple non-sulfur (PNS) bacteria may be one of the most attractive candidates for PHA production improvements due to their unique characteristics. PNS bacteria have diverse growth modes such as aerobic respiratory, anaerobic photoheterotrophic, and photolithoautotrophic growth, and can assimilate various kinds of carbon sources including sugars and organic acids. Therefore, PNS bacteria may be able to respond to various types of growth requests depending on the situation. In addition, PNS bacteria can utilize light energy for potent adenosine triphosphate synthesis, and thus can efficiently produce various materials such as hydrogen, 5-aminolevulinic acid, and PHA at high yields. Many useful chemicals, such as hydrogen, PHA, and carotenoids, can be produced simultaneously by PNS bacteria [7, 8]. Therefore, PNS bacteria is occasionally chosen as a host microorganism for PHA production [9–14]. Suzuki et al. [9] reported that when the *Rhodobacter sphaeroides* RV strain was grown under anaerobic, photoheterotrophic, and pH–stat conditions, the RV strain grown at weak alkaline pH (8.0 or 8.5) produced more PHB than those grown at neutral pH (7.0 or 7.5). Previously, various other growth factors were also examined such as types of substrates and light intensity [10], and this study revealed that *R. sphaeroides* ATCC 17023 strain preferred a carbon source of about 100 mM acetate for PHB production and more than 30 μE m^−2^ s^−1^ of light intensity for anaerobic photoheterotrophic growth. Similarly, acetate was often reported as a preferred carbon source for PHB production by other PNS bacteria [12–14]. Hassan et al. [11] evaluated the two-stage PHA production from palm oil mill effluent using palm oil sludge and *R. sphaeroides* IFO 12203 strain. Formic acid is released when palm oil is treated using sludge in the first stage, which drastically inhibits PHA production by *R. sphaeroides* IFO 12203 in the second stage. Sangkharak et al. isolated halotolerant *R. sphaeroides* E16 strain from a marine source and created its mutant strains, N20 and U7, by using mutation inducing treatments [13, 14]. The obtained N20 strain finally produced about 2.5-fold higher PHB (7.8 g l^−1^) than the wild type strain through growth optimization. However, genetic differences between the wild type strain and each of the mutant strains were not analyzed.

However, despite the attractive characteristics of PNS bacteria as a host microorganism for bioproduction, only a few studies about PHA including poly (3-hydroxybutyrate) (PHB) biosynthesis using genetic engineering techniques have been reported so far [15–18]. Furthermore, these studies did not focus on improving PHA production, but on describing the genetic function and PHA biosynthesis in PNS bacteria. In other words, studies aimed at improving PHA production by PNS bacteria using genetic engineering have not been reported yet.

Generally, PHA biosynthesis is promoted in carbon rich and other nutrient efficient condition [19]. Therefore, when PHA is produced from wastes containing various kinds of nutrients such as nitrogen, this nutrient rich environment may lead to a poor PHA yield. Hence, to efficiently produce PHA from wastes, purification procedures such as denitrification are needed. However, these procedures are often troublesome and costly. To overcome this problem, the development of genetic engineering approaches for the biosynthesis of PHA is important.

**Fig. 1:**
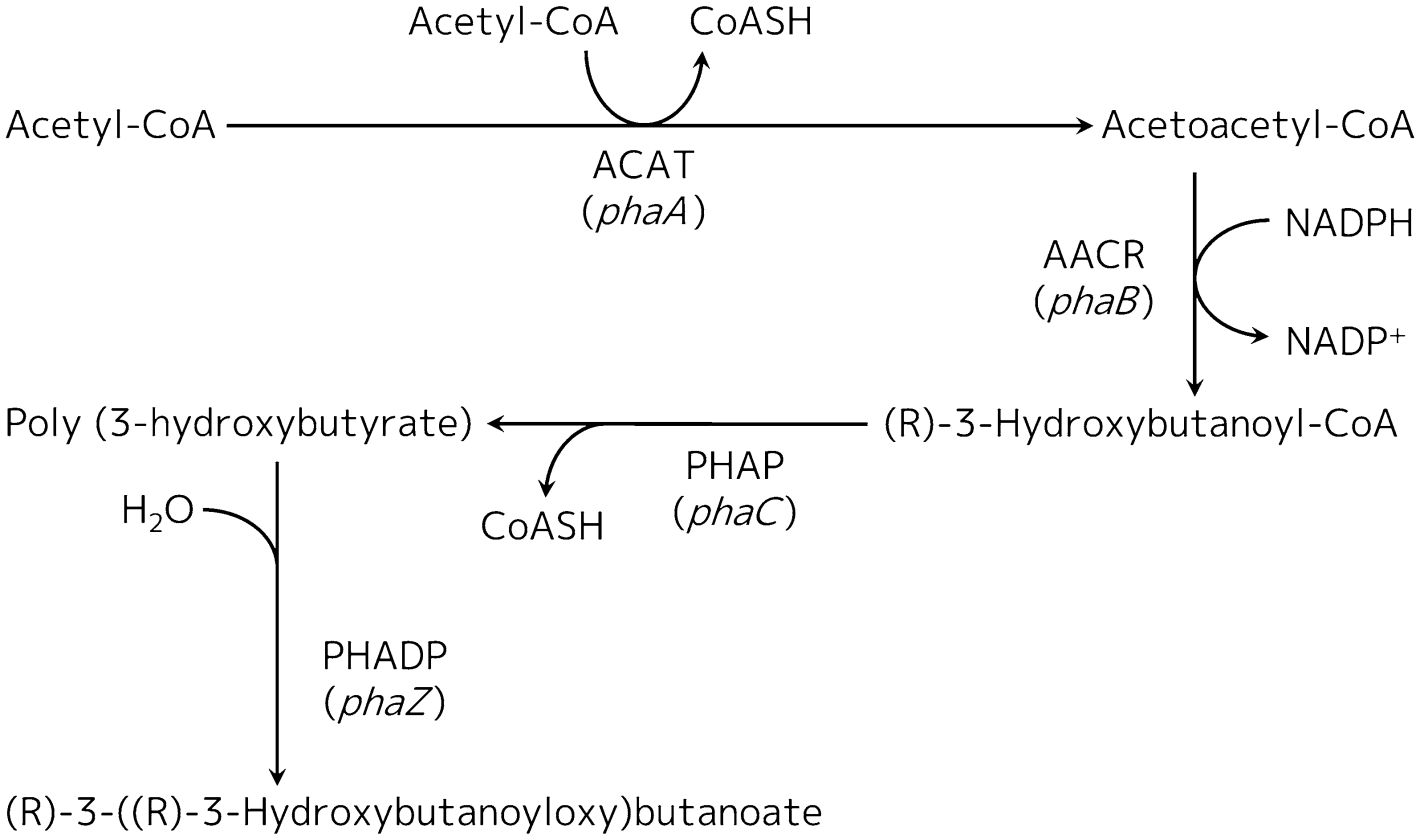
PHB biosynthetic pathway. First, ACAT transfers an acetyl group from one molecule of acetyl-CoA to the acetyl group of a second acetyl-CoA and produces acetoacetyl-CoA and CoASH. Second, AACR reduces the acetoacetyl group of acetoacetyl-CoA and produces (R)-3-hydroxybutanoyl-CoA. Finally, PHAP polymerizes several (R)-3-hydroxybutanoyl groups from (R)-3-hydroxybutanoyl-CoA molecules and produces PHB and CoASH. Synthesized PHB is degraded by PHADP to form (R)-3-((R)-3-hydroxybutanoyloxy) butanoate

Therefore, we aimed to produce PHB by genetic engineering using *R. sphaeroides* as a host microorganism. PHB was synthesized from acetyl coenzyme A (acetyl-CoA) via catalytic reactions of three kinds of enzymes (Fig. 1). First, acetyl coenzyme A acetyltransferase (enzyme: ACAT, gene: *phaA*) transfers an acetyl group from one molecule of acetyl-CoA to the acetyl group of a second acetyl-CoA and produces acetoacetyl coenzyme A (acetoacetyl-CoA) and coenzyme A (CoASH). Second, acetoacetyl coenzyme A reductase (enzyme: AACR, gene: *phaB*) reduces the acetoacetyl group of acetoacetyl-CoA and produces (R)-3-hydroxybutanoyl coenzyme A ((R)-3-hydroxybutanoyl-CoA) using nicotinamide adenine dinucleotide phosphate (NADPH) as a cofactor. Finally, poly (3-hydroxyalkanoate) polymerase (enzyme: PHAP, gene: *phaC*) polymerizes several (R)-3-hydroxybutanoyl groups from (R)-3-hydroxybutanoyl-CoA molecules and produces PHB and CoASH. Synthesized PHB is degraded by poly (3-hydroxyalkanoate) depolymerase (enzyme: PHADP; gene: *phaZ*) as necessary, and used as a carbon source.

First, we searched for the PHB biosynthetic genes in *R. sphaeroides* 2.4.1 genome database and found that it harbors four genes annotated as *phaA* (KEGG ID: RSP_0745, RSP_1354, RSP_2197, and RSP_3184); two as *phaB* (KEGG ID: RSP_0747 and RSP_3963), and two more as *phaC* (KEGG ID: RSP_0382 and RSP_1257). The function of most of these genes has not been characterized yet.

In this study, to improve PHB yield in *R. sphaeroides*, we constructed a *phaZ* (KEGG ID: RSP_0383)-disrupted strain and several strains overexpressing PHB biosynthetic genes to evaluate their effect in the synthesis of the polymer by *R. sphaeroides* cells.

## Results

### PHB production of *R. sphaeroides* HJ (pLP-1.2) and HJΔ*phaZ* (pLP-1.2) strains

**Fig. 2:**
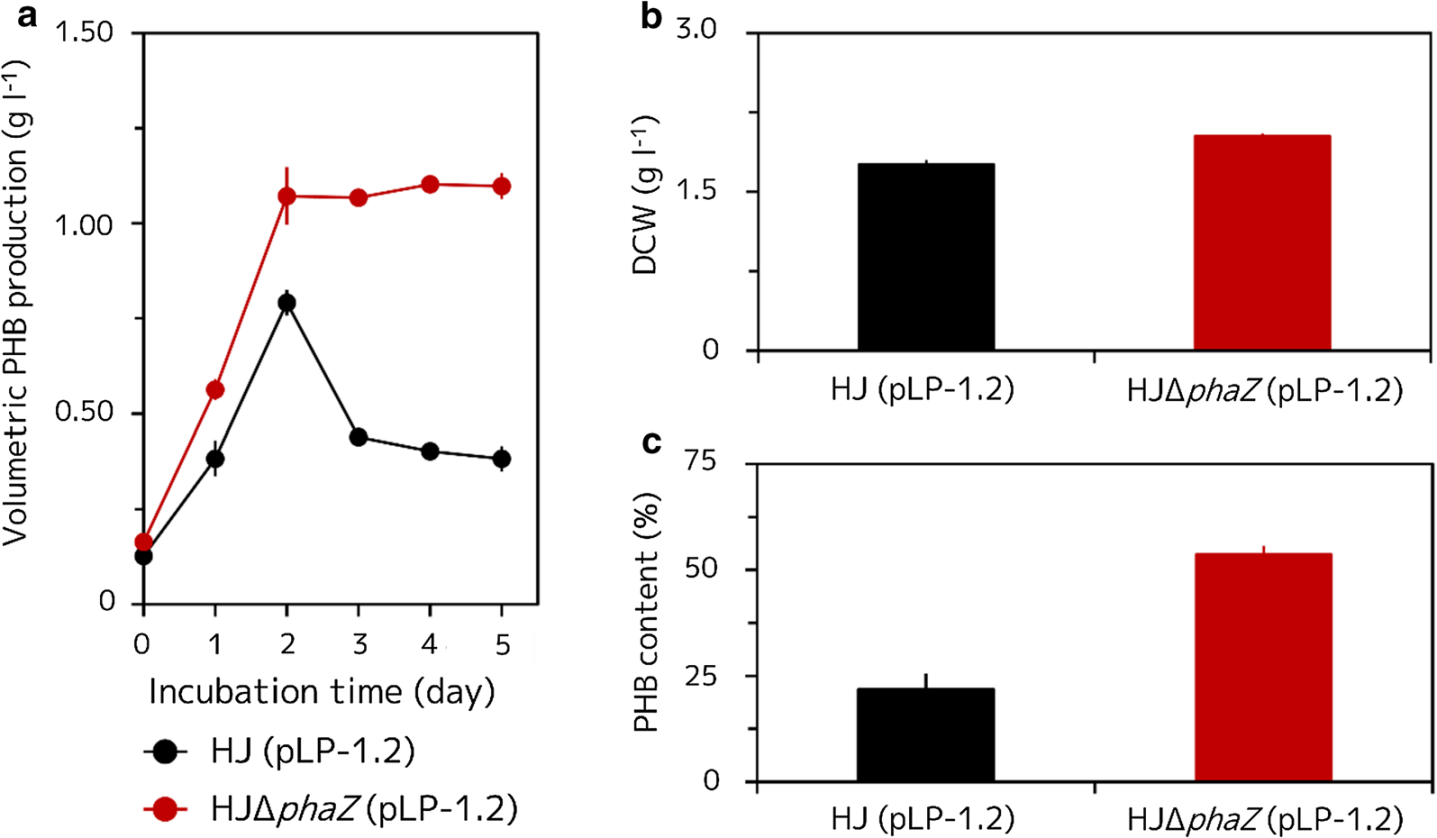
Volumetric PHB production, DCW, and PHB content of the HJ (pLP-1.2) and HJΔ*phaZ* (pLP-1.2) strains. **a** Volumetric PHB production during a 5 day culturing time; **b** DCW after 5 days of incubation; **c** PHB content after 5 days of incubation. Cells were grown anaerobically at 30 °C under illumination at 8.2 W m^−2^ for 5 days. Three independent fermentation experiments were done. Data are presented as the mean ± standard deviation (n = 3)

The volumetric PHB production of *R. sphaeroides* HJ (pLP-1.2) (*R. sphaeroides* HJ harboring intact pLP-1.2) and HJΔ*phaZ* (pLP-1.2) (*phaZ* disrupted *R. sphaeroides* HJ harboring intact pLP-1.2) strains was measured. The PHB production of the HJΔ*phaZ* (pLP-1.2) strain was greatly improved compared to that of its parental strain HJ (pLP-1.2), because PHB was not degraded in the mutant strain after 3 days of incubation, while the polymer was degraded in the HJ (pLP-1.2) strain cells (Fig. 2a). In addition, PHB production until 2 days was higher in the HJΔ*phaZ* (pLP-1.2) strain than in the HJ (pLP-1.2) strain. Overall, the HJΔ*phaZ* (pLP-1.2) strain showed about 2.9-fold higher volumetric PHB production (1.10 ± 0.03 g l^−1^) than that of the HJ (pLP-1.2) strain (0.38 ± 0.03 g l^−1^) after 5 days of incubation by losing its PHB degrading ability. Interestingly, the dry cell weight (DCW) of the HJΔ*phaZ* (pLP-1.2) strain showed only a slight increase (2.02 ± 0.01 g l^−1^), when compared to the HJ (pLP-1.2) strain (1.76 ± 0.04 g l^−1^), whereas the percentage of PHB content was much higher in the HJΔ*phaZ* (pLP-1.2) strain (54.2 ± 3.6%) than in its parent strain HJ (pLP-1.2) (21.8 ± 3.6%) after 5 days of incubation.

### PHB production of recombinant *R.sphaeroides* HJΔ*phaZ* strains overexpressing PHB biosynthetic genes

The *R. sphaeroides* HJΔ*phaZ* strain was used as the host strain for further improvement of PHB production by overexpressing PHB biosynthetic genes. Crude extracts of the eight recombinant strains showed higher ACAT, AACR, and PHAP activities than those of the parent strain, indicating successful expression of these genes and production of their corresponding products in the bacterial cells (Table 1).

**Table 1:** ACAT, AACR, and PHAP activities in recombinant *R. sphaeroides* HJ cells

Strain	Enzyme activity (U/g-protein)		
	ACAT	AACR	PHAP
HJΔ*phaZ* (pLP-1.2)	8.2 ± 0.3	5.3 ± 0.2	0.7 ± 0.1
HJΔ*phaZ* (*phaA1*)	22.5 ± 0.6	–	–
HJΔ*phaZ* (*phaA2*)	18.6 ± 0.8	–	–
HJΔ*phaZ* (*phaA3*)	19.2 ± 0.5	–	–
HJΔ*phaZ* (*phaA4*)	19.1 ± 0.5	–	–
HJΔ*phaZ* (*phaB1*)	–	19.5 ± 1.2	–
HJΔ*phaZ* (*phaB2*)	–	21.0 ± 1.7	–
HJΔ*phaZ* (*phaC1*)	–	–	4.8 ± 0.1
HJΔ*phaZ* (*phaC2*)	–	–	3.4 ± 0.2
HJΔ*phaZ* (*phaA3*/*phaB2*/*phaC1*)	20.5 ± 1.5	16.0 ± 0.5	3.5 ± 0.3

The recombinant *R. sphaeroides* HJ cells were anaerobically grown in AAY medium containing 10 mM AS at 30 °C for 3 days under illumination at 8.2 W m^−2^

–, The data were not measured

**Fig. 3:**
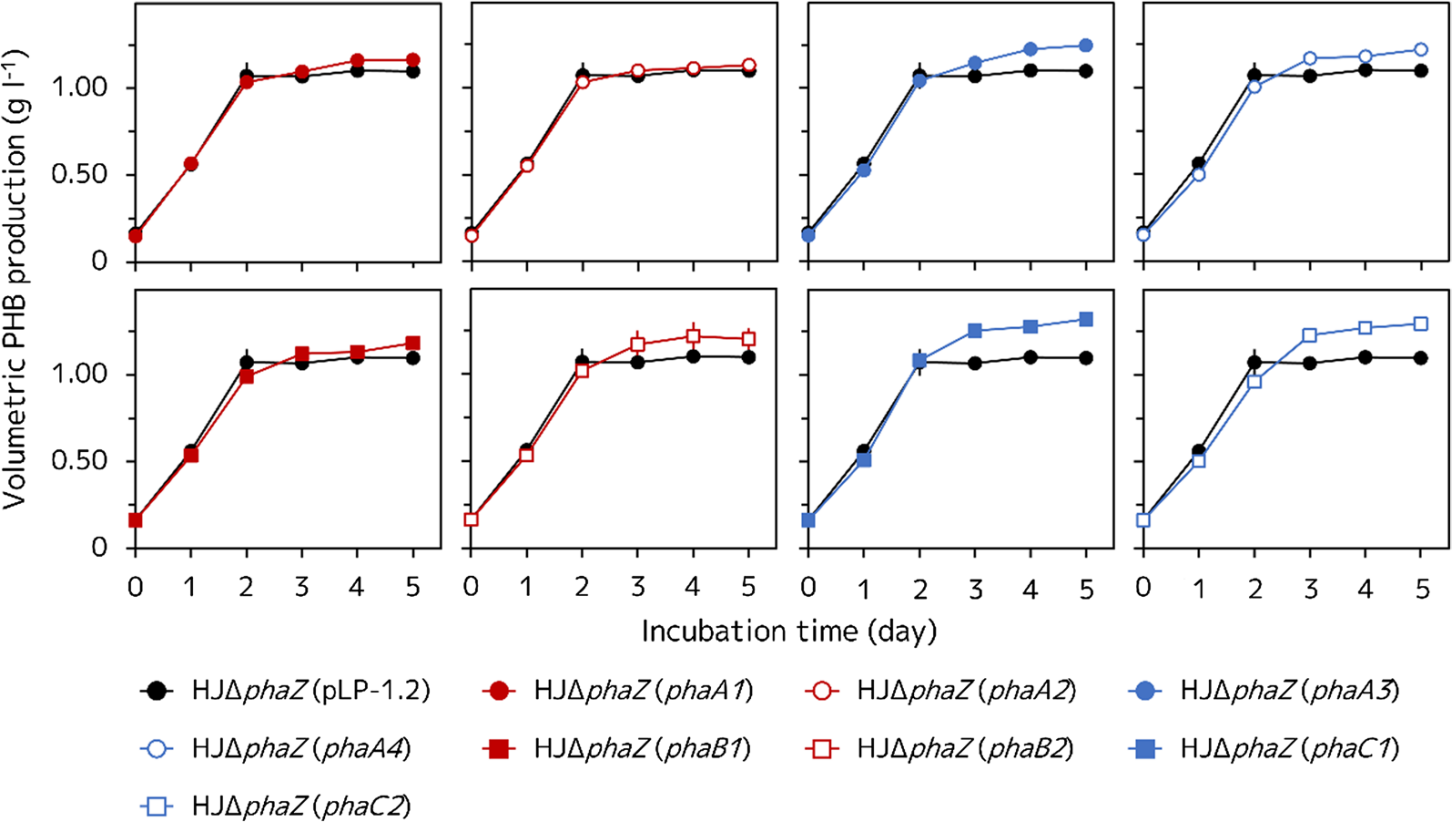
Volumetric PHB production of the recombinant HJΔ*phaZ* strains. Cells were anaerobically grown at 30 °C under illumination at 8.2 W m^−2^ for 5 days. Three independent fermentation experiments were done. Data are presented as the mean ± standard deviation (n = 3)

Then, we compared the PHB production of the four recombinant HJΔ*phaZ* strains overexpressing each one of the *phaA* genes with that of the parental strain HJΔ*phaZ* (pLP-1.2). We found that after 5 days of incubation, the HJΔ*phaZ* (*phaA1*) (*R. sphaeroides* HJΔ*phaZ* overexpressing *phaA1*) strain had produced PHB at a slightly higher concentration (1.16 ± 0.01 g l^−1^) than the parental strain (1.10 ± 0.03 g l^−1^), while both the HJΔ*phaZ* (*phaA3*) (*R. sphaeroides* HJΔ*phaZ* overexpressing *phaA3*) and HJΔ*phaZ* (*phaA4*) (*R. sphaeroides* HJΔ*phaZ* overexpressing *phaA4*) strains had apparently produced more PHB (1.25 ± 0.01 and 1.22 ± 0.01 g l^−1^, respectively) than the HJΔ*phaZ* (pLP-1.2) strain (Fig. 3). However, the HJΔ*phaZ* (*phaA2*) (*R. sphaeroides* HJΔ*phaZ* overexpressing *phaA2*) strain showed almost the same PHB production profile (1.13 ± 0.02 g l^−1^ at 5 days of incubation) compared to the HJΔ*phaZ* (pLP-1.2) strain at each one of the time points evaluated; whereas the crude extract from the HJΔ*phaZ* (*phaA2*) strain cells exhibited a higher ACAT activity than that from the HJΔ*phaZ* (pLP-1.2) strain cells, as previously mentioned (Table 1).

**Fig. 4:**
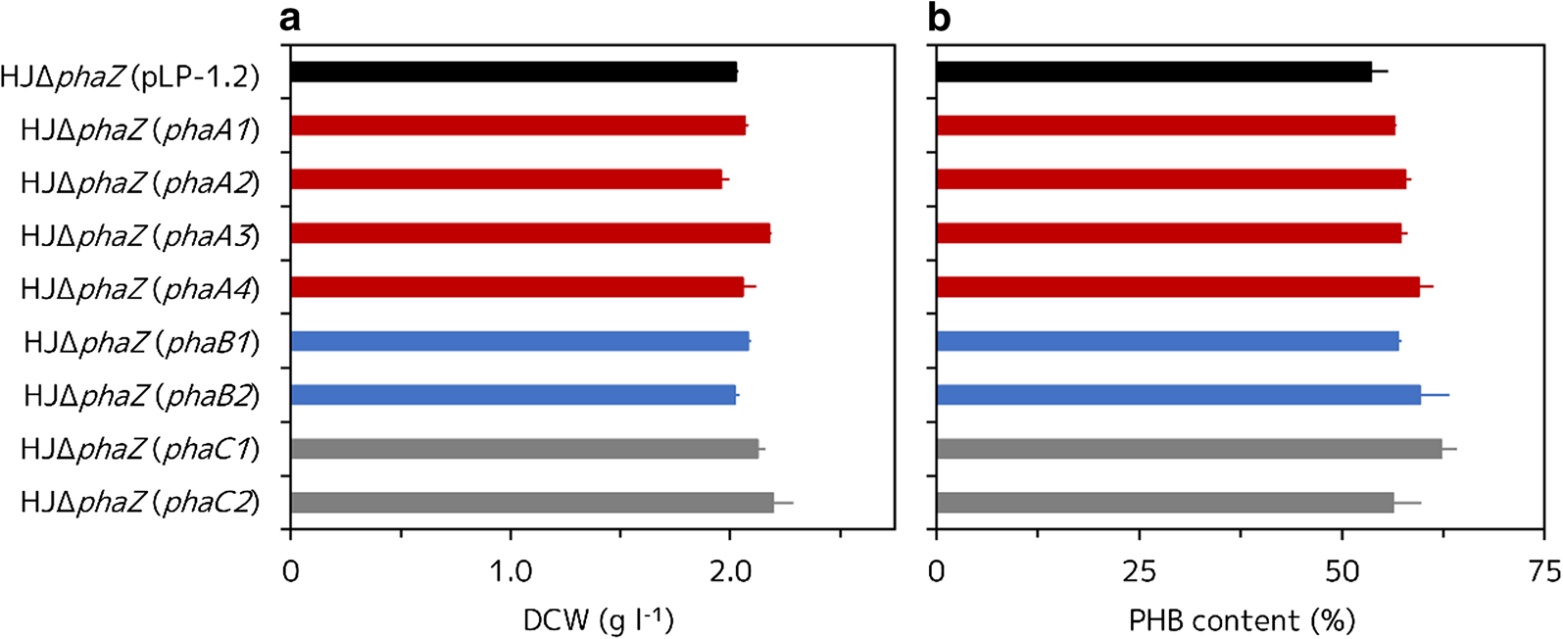
Dry cell weight and PHB content of recombinant HJΔ*phaZ* strains. **a** DCW of each recombinant HJΔ*phaZ* strain after 5 days of incubation. **b** PHB content of each recombinant HJΔ*phaZ* strain after 5 days of incubation. Three independent fermentation experiments were done. Data are presented as the mean ± standard deviation (n = 3)

Interestingly, these strains showed somewhat different results in DCW and PHB content compared to the volumetric PHB production. The HJΔ*phaZ* (*phaA3*) strain showed a slightly higher DCW (2.18 ± 0.01 g l^−1^) than the HJΔ*phaZ* (pLP-1.2) strain (2.02 ± 0.01 g l^−1^) after 5 days of incubation (Fig. 4a), while the HJΔ*phaZ* (*phaA1*), HJΔ*phaZ* (*phaA2*), and HJΔ*phaZ* (*phaA4*) strains showed almost the same or even a slightly decreased DCW (2.02 ± 0.01, 1.96 ± 0.04, and 2.06 ± 0.06 g l^−1^, respectively) compared to the HJΔ*phaZ* (pLP-1.2) strain; whereas the HJΔ*phaZ* (*phaA4*) stain apparently produced a higher volumetric PHB than that of the HJΔ*phaZ* (pLP-1.2) strain. In contrast, the PHB content of the HJΔ*phaZ* (*phaA1*), HJΔ*phaZ* (*phaA2*), HJΔ*phaZ* (*phaA3*) and HJΔ*phaZ* (*phaA4*) strains was higher (56.5 ± 0.2, 57.8 ± 0.7, 57.2 ± 0.9, and 59.4 ± 1.8% after 5 days of incubation, respectively) than that of the HJΔ*phaZ* (pLP-1.2) strain (54.2 ± 3.6%) (Fig. 4b).

On the other hand, both of the HJΔ*phaZ* (*phaB1*) (*R. sphaeroides* HJΔ*phaZ* overexpressing *phaB1*) and HJΔ*phaZ* (*phaB2*) (*R. sphaeroides* HJΔ*phaZ* overexpressing *phaB2*) stains produced more PHB (1.18 ± 0.01 and 1.20 ± 0.06 g l^−1^) than the control HJΔ*phaZ* (pLP-1.2) strain after 5 days of incubation (Fig. 3). These two strains showed almost the same DCW (2.08 ± 0.02 and 2.02 ± 0.02 g l^−1^) (Fig. 4a) and a higher PHB content (56.9 ± 0.4 and 59.6 ± 3.6%) (Fig. 4b) than those of the HJΔ*phaZ* (pLP-1.2) strain after incubation of 5 days.

Overexpression of the *phaC* gene also improved PHB production, more efficiently than the overexpression of either *phaA* or *phaB* genes. Both the HJΔ*phaZ* (*phaC1*) (*R. sphaeroides* HJΔ*phaZ* overexpressing *phaC1*) and HJΔ*phaZ* (*phaC2*) (*R. sphaeroides* HJΔ*phaZ* overexpressing *phaC2*) strains produced more PHB (1.32 ± 0.02 and 1.29 ± 0.02 g l^−1^) (Fig. 3), and showed a higher DCW (2.12 ± 0.04 and 2.19 ± 0.09 g l^−1^) (Fig. 4a) and PHB content (62.2 ± 1.9 and 56.3 ± 3.5%) than the parental strain (Fig. 4b), after 5 days of incubation.

### PHB production of *R. sphaeroides* HJΔ*phaZ* (*phaA3*/*phaB2*/*phaC1*) strain at different concentrations of ammonium sulfate

From our results regarding PHB production of the recombinant HJΔ*phaZ* strains overexpressing the predicted PHB biosynthetic genes, and because volumetric PHB production is the most important parameter for industrial mass production of PHB, we decided to overexpress *phaA3*, *phaB2*, and *phaC1* genes simultaneously in the HJΔ*phaZ* strain. The HJΔ*phaZ* (*phaA3*/*phaB2*/*phaC1*) (*R. sphaeroides* HJΔ*phaZ* overexpressing *phaA1*, *phaB2*, and *phaC1*) strain and the control HJ (pLP-1.2) and HJΔ*phaZ* (pLP-1.2) strains were grown in AAY medium containing various concentration of ammonium sulfate (AS) as nitrogen source.

**Fig. 5:**
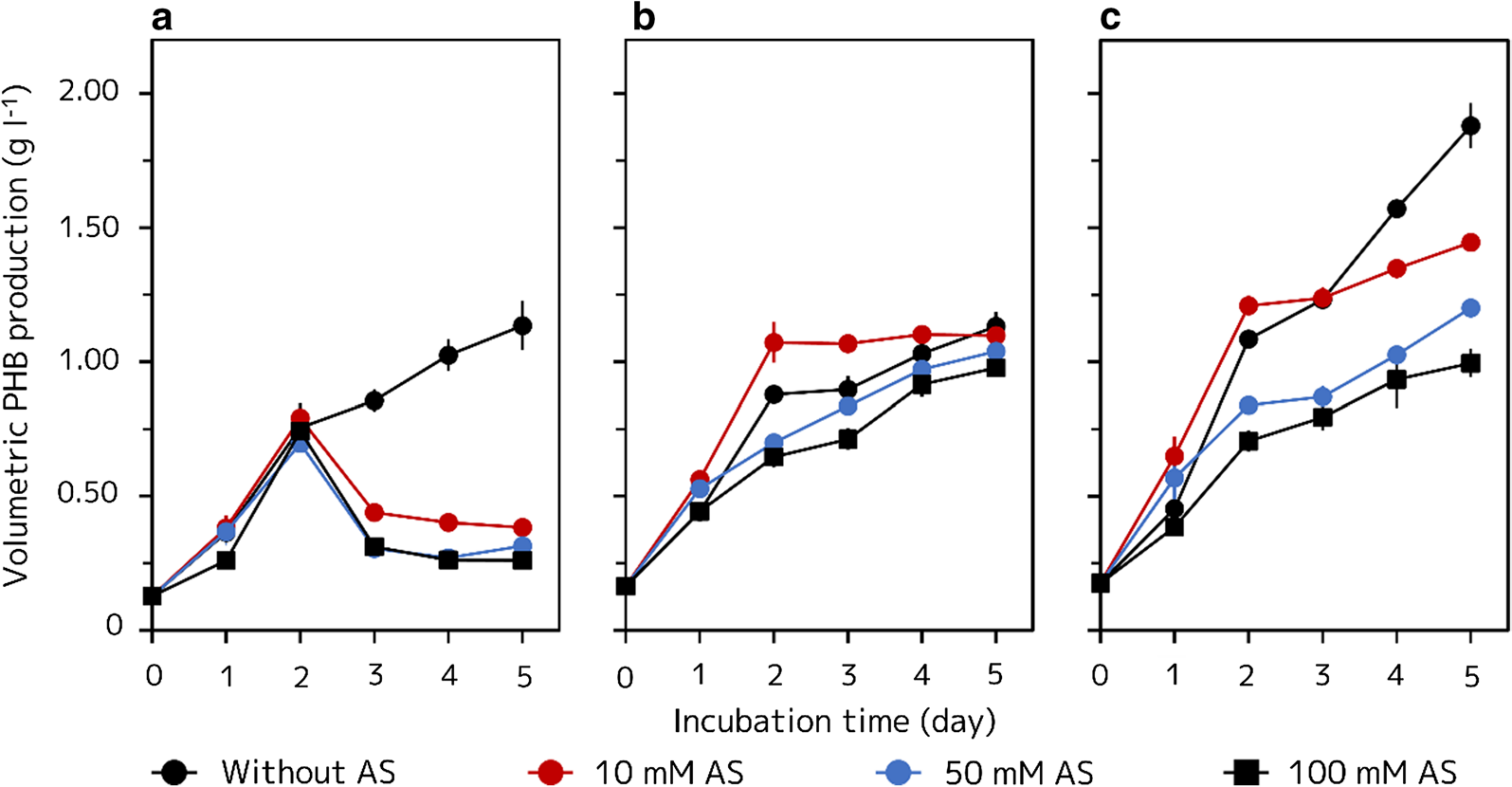
Volumetric PHB production of the recombinant HJ and HJΔ*phaZ* strains at different AS concentrations. **a** HJ (pLP-1.2) strain grown in AAY medium with various concentrations of AS. **b** HJΔ*phaZ* (pLP-1.2) strain grown in AAY medium with various concentrations of AS. **c** HJΔ*phaZ* (*phaA3*/*phaB2*/*phaC1*) strain grown in AAY medium with various concentrations of AS. Cells were grown anaerobically at 30 °C under illumination at 8.2 W m^−2^ for 5 days. Three independent fermentation experiments were done. Data are presented as the mean ± standard deviation (n = 3)

**Fig. 6:**
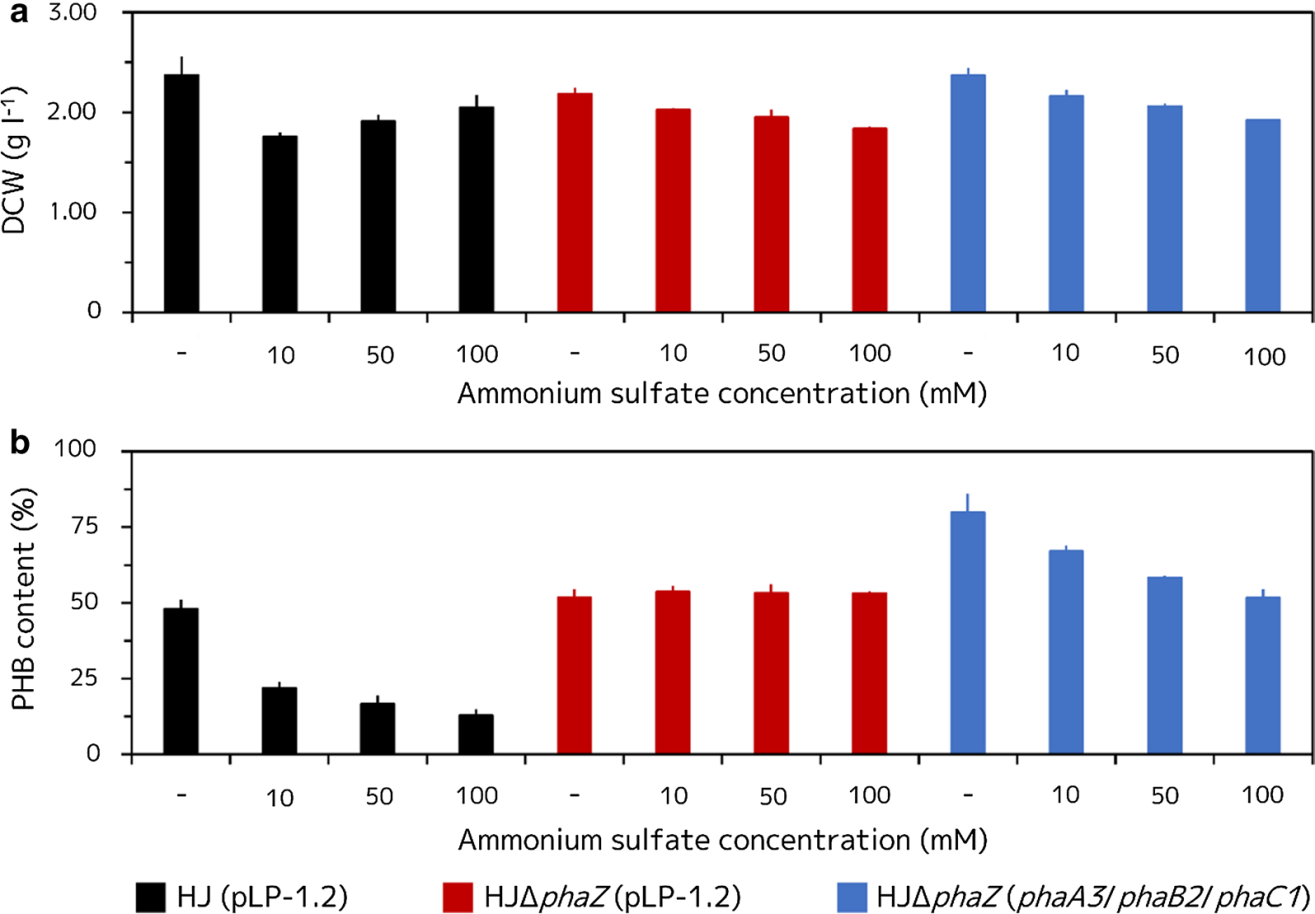
DCW and PHB content of the recombinant HJ and HJΔ*phaZ* strains at different AS concentrations. **a** DCW of each of the recombinant HJ and HJΔ*phaZ* strains grown in AAY medium with various concentrations of AS after 5 days of incubation. **b** PHB content of each of the recombinant HJ and HJΔ*phaZ* strains grown in AAY medium with various concentrations of AS after 5 days of incubation. Cells were grown anaerobically at 30 °C under illumination at 8.2 W m^−2^ for 5 days. Three independent fermentation experiments were done. Data are presented as the mean ± standard deviation (n = 3)

In AAY medium without AS, the HJ (pLP-1.2) strain continuously accumulated PHB (1.13 ± 0.19 g l^−1^ after 5 days of incubation) (Fig. 5a). In contrast, when the HJ (pLP-1.2) strain was grown in AAY medium containing AS, intracellular PHB was degraded after 3 days of incubation; PHB degradation tended to increase at higher concentration of AS (10 mM AS: 0.38 ± 0.03, 50 mM AS: 0.31 ± 0.04, and 100 mM AS: 0.26 ± 0.03 g l^−1^ after 5 days of incubation, respectively) (Fig. 5a). The DCW and associated PHB content of the HJ (pLP-1.2) strain were also affected by AS concentration (DCW; without AS: 2.37 ± 0.19, 10 mM AS: 1.76 ± 0.04, 50 mM AS: 1.91 ± 0.07, and 100 mM AS: 2.05 ± 0.13 g l^−1^ after 5 days of incubation, respectively; PHB content; without AS: 48.0 ± 2.9, 10 mM AS: 21.8 ± 2.1, 50 mM AS: 16.6 ± 2.7, and 100 mM AS: 12.8 ± 2.0% after 5 days of incubation, respectively) (Fig. 6a, b).

On the other hand, the HJΔ*phaZ* (pLP-1.2) strain continuously accumulated PHB even when the strain was grown in AAY medium containing high concentration of AS (Fig. 5b). Though the volumetric PHB production of the HJΔ*phaZ* (pLP-1.2) strain was apparently affected by the concentration of AS, the volumetric PHB production after 5 days of incubation was similar in each AS concentration (without AS: 1.13 ± 0.05, 10 mM AS: 1.10 ± 0.03, 50 mM AS: 1.04 ± 0.05 and 100 mM AS: 0.98 ± 0.02 g l^−1^ after 5 days of incubation, respectively). These different PHB accumulation profiles during the 5 days of cultivation may be caused by cell growth. DCW of the HJΔ*phaZ* (pLP-1.2) strain grown at different AS concentration decreased as concentration increased (without AS: 2.18 ± 0.05, 10 mM AS: 2.02 ± 0.01, 50 mM AS: 1.95 ± 0.08 and 100 mM AS: 1.84 ± 0.01 g l^−1^ after 5 days of incubation, respectively) (Fig. 6a), whereas PHB content of the HJΔ*phaZ* (pLP-1.2) strain was not changed by AS concentration (without AS: 51.9 ± 2.5, 10 mM AS: 53.6 ± 2.1, 50 mM AS: 53.3 ± 2.7 and 100 mM AS: 53.2 ± 0.6% after 5 days of incubation, respectively) (Fig. 6b). Therefore, PHB accumulation of the HJΔ*phaZ* (pLP-1.2) strain was not affected by AS concentration, however the cell growth of the strain decreased by the presence of AS, and, consequently, its volumetric PHB production also decreased.

The HJΔ*phaZ* (*phaA3*/*phaB2*/*phaC1*) strain produced more PHB than the HJ (pLP-1.2) and the HJΔ*phaZ* (pLP-1.2) strains (Fig. 5c). When the HJΔ*phaZ* (*phaA3*/*phaB2*/*phaC1*) strain was grown in AAY medium without AS, the strain showed a significantly high PHB accumulation (1.88 ± 0.08 g l^−1^ after 5 days of incubation). However, this significant PHB accumulation was suppressed in presence of AS, even when only 10 mM AS was added to AAY media (10 mM AS: 1.45 ± 0.03, 50 mM AS: 1.20 ± 0.01, 100 mM AS: 0.99 ± 0.05 g l^−1^ after 5 days of incubation). When the HJΔ*phaZ* (*phaA3*/*phaB2*/*phaC1*) strain was grown in AAY medium containing 100 mM AS, PHB accumulation by simultaneous overexpression of *phaA3*, *phaB2* and *phaC1* was almost completely inhibited, because the volumetric PHB production profiles in AAY media containing 100 mM AS of the HJΔ*phaZ* (*phaA3*/*phaB2*/*phaC1*) and HJΔ*phaZ* (pLP-1.2) strains were almost the same. Unlike the PHB content of the HJΔ*phaZ* (pLP-1.2), the HJΔ*phaZ* (*phaA3*/*phaB2*/*phaC1*) showed decreased PHB content depending on AS concentration (without AS: 79.8 ± 6.0, 10 mM AS: 67.0 ± 1.9, 50 mM AS: 58.4 ± 0.5, and 100 mM AS: 51.7 ± 2.7% after 5 days of incubation, respectively) (Fig. 5c). These results also indicate that the presence of AS in AAY medium suppresses PHB biosynthesis. DCW of the HJΔ*phaZ* (*phaA3*/*phaB2*/*phaC1*) strain also showed a growth inhibition induced by high concentrations of AS (without AS: 2.37 ± 0.08, 10 mM AS: 2.16 ± 0.06, 50 mM AS: 2.06 ± 0.03 and 100 mM AS: 1.92 ± 0.01 g l^−1^ after 5 days of incubation, respectively) (Fig. 6a).

**Fig. 7:**
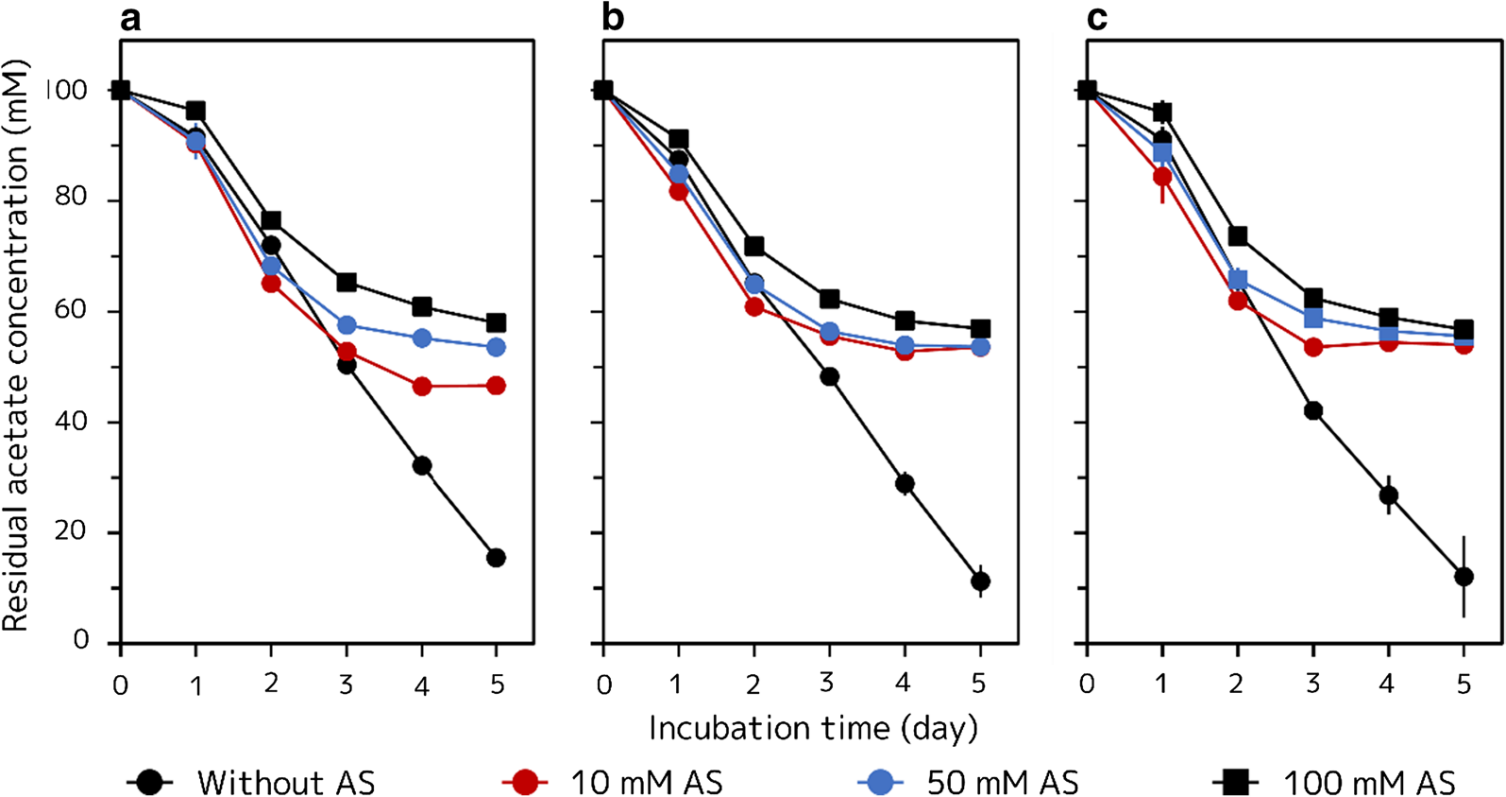
Residual acetate concentration in the media during PHB production of recombinant HJ and HJΔ*phaZ* strains at different AS concentrations. **a** HJ (pLP-1.2) strain grown in AAY medium with various concentrations of AS. **b** HJΔ*phaZ* (pLP-1.2) strain grown in AAY medium with various concentrations of AS. **c** HJΔ*phaZ* (*phaA3*/*phaB2*/*phaC1*) strain grown in AAY medium with various concentrations of AS. Cells were anaerobically grown at 30 °C under illumination at 8.2 W m^−2^ for 5 days. Three independent fermentation experiments were done. Data are presented as the mean ± standard deviation (n = 3)

Residual acetate concentrations in AAY medium may also provide worthwhile information. When recombinant strains were grown in AAY media without AS, the cells continuously consumed acetate through the 5 days of culture (Fig. 7a–c). In contrast, addition of AS to AAY medium lead to decreased acetate consumptions. Furthermore, two *phaZ* disrupted strains showed slightly increased acetate consumptions compared to the HJ (pLP-1.2) strain in AAY media without AS, whereas these two strains showed decreased acetate consumption compared to the parent strain in AAY medium containing AS. The PHB production profiles of the HJΔ*phaZ* (pLP-1.2) and HJΔ*phaZ* (*phaA3*/*phaB2*/*phaC1*) strains were much different, especially when cells were grown in AAY media without AS. However, the acetate consumption profiles of these two strains were very similar.

## Discussion

In this study, we found eight genes presumed to be involved in PHB biosynthesis in the genome of *R. sphaeroides* 2.4.1, and showed that all of their gene products exhibited annotated enzyme activities (Table 1). Also, we demonstrated that many of them enhance PHB production when overexpressed (Fig. 3). Furthermore, we revealed that the disruption of *phaZ* also improved PHB production (Fig. 2a), especially under nitrogen rich condition (Fig. 5) due to the loss of its PHB degrading ability. This fact shows that the gene product of *phaZ* has PHADP activity as annotated. Generally, PHB degradation in the presence of nitrogen is observed in various other organisms, and therefore many studies on PHB production have been carried out in nitrogen limited conditions [9–14, 21, 24–26]. However, when *phaZ* is disrupted, the recombinant strain is considered to be not able to degrade PHB due to the lack of PHADP activity, even when the medium contains nitrogen, and consequently, PHB continues to be accumulated in cells. Deletion or disruption of *phaZ* have often employed for improving PHA production in other microorganisms [21, 24–26], and all of these attempts have resulted in improved PHA production. However, these studies were carried out under nitrogen limited conditions. Therefore, we have first demonstrated the effectiveness of *phaZ* gene disruption for enhancing PHB production under nitrogen rich conditions.

In this study, we further improved PHB production in *R. sphaeroides* HJ by the simultaneous overexpression of *phaA3*, *phaB2*, and *phaC1* genes (Fig. 5). However, the PHB yield achieved in this study is lower than those from other PHB producing microorganisms such as *Cupriavidus necator* [6, 20, 21]. These microorganisms are often grown aerobically in sugars and oils as carbon sources. In contrast, in this study, *R. sphaeroides* strains were anaerobically and photoheterotrophically grown in a medium with an organic acid of acetate as the carbon source. Therefore, comparisons between other PHB producing microorganisms and *R. sphaeroides* are difficult. Indeed, *R. sphaeroides* grown in anaerobic and photoheterotrophic conditions apparently showed decreased DCW and PHB content compared to the cells grown in aerobic conditions [14]. In addition, optimization of growth condition such as examination of growth temperature and medium composition may further improve cell proliferation and its associated PHB production in *R. sphaeroides*. Indeed, some growth optimization studies practically improved the PHB yields by PNS bacteria [10, 14]. Furthermore, the characteristics of PNS bacteria mentioned above may enable us to produce PHB from wastes containing various kinds of organic compounds as nutrients, in warm outdoor conditions, and with a lot of sunlight. Our findings may lead to the development of an efficient and environmentally friendly PHB production protocol.

Unfortunately, PHB production by overexpression of genes encoding PHB synthetic enzymes was highly suppressed by the presence of nitrogen in the medium (Fig. 5c). Kranz et al. previously reported that genes involved in PHB biosynthesis in *R. capsulatus* were constantly expressed even when cells were grown in the presence of nitrogen [15]. Therefore, the cause of the suppression of PHB biosynthesis by nitrogen may be a posttranscriptional process. The gene expression vector pLP-1.2 used in this study has a *puf* promoter containing part of the bacteriochlorophyll biosynthetic and light harvesting machinery genes [22] and therefore, can highly express the gene of interest not only during anaerobic photosynthesis but also during aerobic respiration [23]. Indeed, successful production of each gene product was confirmed by assaying its activity (Table 1) even when the recombinant strains used for the enzyme assays were grown in AAY medium containing 10 mM AS. The fact that nitrogen may regulate PHB production post transcriptionally, together with the results showing that gene products activities can be detected even when the recombinant strains were grown under nitrogen rich conditions suggest that improvement of PHB yield under nitrogen rich conditions by genetic engineering may be a hard problem.

In the HJΔ*phaZ* (pLP-1.2) strain cells, PHAP activity in crude extract was much lower than that of ACAT and AACR (Table 1). At first glance, this result indicates that PHAP, encoded by *phaC*, is the limiting step for PHB biosynthesis in *R. sphaeroides* HJ cells. However, not only the overexpression of *phaC* genes, but also the overexpression of *phaA* and *phaB* genes improved PHB accumulation in the *R. sphaeroides* HJΔ*phaZ* (pLP-1.2) strain (Fig. 3). If PHAP were the limiting step in PHB biosynthesis in *R. sphaeroides* HJ cells, the overexpression of *phaA* and *phaB* genes should not improve PHB yield. In other organisms, there are several reports that *phaB* may be the limiting step for PHB biosynthesis [27]; results obtained in this study do not agree with these reports. Generally, enzyme activities are easily changed by various factors such as substrate supply and the presence of inhibitors. Therefore, the limiting step for PHB biosynthesis in *R. sphaeroides* HJ cells may also change by these various factors. Our results showing that overexpression of *phaA*, *phaB*, and *phaC* genes increased PHB production in *R. sphaeroides* HJ cells may be due to changes in the substrate supply by the overexpression of these genes themselves. Clarifying these molecular biological mechanisms may be very difficult. However, in this study, the fact that overexpression of *phaA*, *phaB*, and *phaC* genes is one of the effective strategies to improve PHB yield in *R. sphaeroides* was revealed.

We observed interesting results regarding the DCW and PHB concentrations when each strain was cultured under various concentrations of AS (Fig. 6a, b). Although the DCW from the HJΔ*phaZ* (pLP-1.2) and HJΔ*phaZ* (*phaA3*/*phaB2*/*phaC1*) strains were decreased in the presence of high concentrations of AS, HJ (pLP-1.2) showed a different profile. Similar to HJΔ*phaZ* (pLP-1.2) and HJΔ*phaZ* (*phaA3*/*phaB2*/*phaC1*) strains, the HJ (pLP-1.2) strain achieved the highest DCW level when the strain was cultured in AAY medium without AS. However, when 10 mM of AS was present in the AAY medium, the DCW of the HJ (pLP-1.2) initially decreased considerably, following which the levels increased with increasing AS concentration. The PHB content of the HJ (pLP-1.2) strain grown in 100 mM AS was very low (12.8 ± 2.0%), therefore, it was considered that most of the DCW production in the HJ (pLP-1.2) strain in the presence of 100 mM AS was caused by the bacterium itself, not including PHB. This fact implies that AS in the medium promoted bacterial growth. Generally, the growth and PHA production of bacteria are significantly affected by C/N ratio (mole of carbon per mole of nitrogen) in the growth medium [28]. Therefore, it is considered that the HJ (pLP-1.2) strain degraded PHB and used it as a carbon source in combination with high concentrations of AS as a nitrogen source. On the other hand, the HJΔ*phaZ* (pLP-1.2) and HJΔ*phaZ* (*phaA3*/*phaB2*/*phaC1*) could not use PHB as carbon source as the *phaZ* genes of these strains were disrupted. Therefore, added AS led to impairment of the C/N ratio in the cells of the strains with disrupted *phaZ* and this impaired C/N ratio may cause poor cell growth in these strains.

Generally, two different loci involved in PHB biosynthesis in bacterial genomes are known [29]. Indeed, *phaA1* and *phaB1*, and *phaC1* and *phaZ* are located on two loci in the *R. sphaeroides* genome. However, other PHB biosynthetic genes in PNS bacteria except for *phaA1*, *phaB1*, and *phaC1* have not been focused so far. Searching for previous PHB biosynthesis studies on genetically engineered PNS bacteria, we found an interesting fact: *R. capsulatus* SB1003 did not lose its PHB biosynthesis ability even after the deletion of the *phaA1* and *phaB1* genes [15]. At first, we suspected that *R. capsulatus* SB1003 could harbor genes corresponding to *phaA2*, *phaA3*, *phaA4*, and *phaB2*. Therefore, we searched for the eight PHB biosynthetic genes of *R. sphaeroides* 2.4.1, overexpressed in this study, in the *R. capsulatus* SB1003 strain genome and found that there are no genes corresponding to *phaB2* nor *phaC2* (Additional file 1: Table S3). Furthermore, no genes annotated as AACR were found except for the *phaB1* corresponding gene. These facts strongly imply that there must be unidentified isozymes of AACR or an alternative PHB biosynthesis pathway in *R. capsulatus* SB1003, like the PHB biosynthesis from fatty acids via the beta-oxidation pathway found in *Pseudomonas* sp. [30]. Thus, unidentified genes encoding PHB biosynthetic enzymes or alternative PHB biosynthesis pathway may also exist in other PNS bacteria.

## Conclusions

In this study, we found eight genes involved in PHB biosynthesis in the genome of *R. sphaeroides* 2.4.1, and revealed that overexpression of these genes increased PHB accumulation in a *R. sphaeroides* HJ strain. In addition, we demonstrated the effectiveness of a *phaZ* disruption to achieve a high PHB accumulation, especially under nitrogen rich conditions. Furthermore, we showed that there is a possibility that PNS bacteria may have other still unidentified genes involved in PHB biosynthesis. Our findings could lead to further improvements of environmentally harmless PHA production methods using PNS bacteria.

## Methods

### Strains and media

*R. sphaeroides* HJ, which was isolated in a previous study [31], and *R. sphaeroides* 2.4.1 were used as a host strain and a source of genes, respectively. *R. sphaeroides* strains were routinely grown at 30 °C in ASY medium consisting of a basal medium (20 mM potassium phosphate buffer (pH 7.0), 1% (v/v) inorganic solution, 1% (v/v) vitamin solution, 0.1 g l^−1^ CaCl_2_·2H_2_O, 0.2 g l^−1^ MgSO_4_·7H_2_O), 10 g l^−1^ disodium succinate, 1.0 g l^−1^ yeast extract, and 1.25 g l^−1^ AS. The composition of both inorganic and vitamin solutions have been previously described [31]. For PHB production, *R. sphaeroides* HJ derivative strains were grown in AAY medium consisting basal medium, 100 mM sodium acetate, 1.0 g l^−1^ yeast extract, and 10 mM AS. *Escherichia coli* NovaBlue (Novagen, Darmstadt, Germany) and S17-1 strains were used for DNA manipulation and conjugation transfer, respectively. *E. coli* strains were aerobically grown at 37 °C in Luria-Bertani (LB) medium (10 g l^−1^ tryptone, 5 g l^−1^ yeast extract, and 10 g l^−1^ sodium chloride). Kanamycin (Km; 20 mg l^−1^), streptomycin (Sm; 20 mg l^−1^), spectinomycin (Spc; 20 mg l^−1^) and sucrose (200 g l^−1^) were added when necessary.

### Construction of plasmids

For construction of the *phaZ* gene disruption vector, *phaZ* and Spc resistance genes (*spc*^*R*^) were amplified by PCR using chromosomal DNA from *R. sphaeroides* HJ and plasmid pHRP311 [32] as templates, with an appropriate set of primers each (Additional file 1: Table S4). The amplified DNA encoding *phaZ* gene was cloned between the *Sph*I and the *Xba*I sites of pK18*mobsacB* [33] to obtain pK18ms-*phaZ*. Subsequently, the amplified DNA encoding *spc*^*R*^ gene was cloned into the *Not*I site of *phaZ* gene in pK18ms-*phaZ* to obtain pK18ms-*phaZ*/*spc*^*R*^.

To construct gene expression vectors, *phaA1*, *phaA2*, *phaA3*, *phaA4*, *phaB1*, *phaB2*, *phaC1* and *phaC2* genes were amplified by PCR using chromosomal DNA of *R. sphaeroides* 2.4.1 as a template, with the appropriate primer sets (summarized in Additional file 1: Table S4). Each PCR product was cloned into the *Xba*I site of pLP-1.2 [34] using the In-Fusion^®^ HD Cloning Kit (TaKaRa Bio, Otsu, Japan) to give pLP-1.2 derivatives. To express *phaA3*, *phaB2* and *phaC1* genes simultaneously in *R. sphaeroides* HJ cells, *phaA3*, *phaB2* and *phaC1* genes were amplified by PCR using primer sets: phaA3F and phaA3R2, phaB2F2 and phaB2R2, and phaC2F2 and phaC2R (Additional file 1: Table S4), using plasmids pLP-*phaA3*, pLP-*phaB2* and pLP-*phaC1* as templates, respectively. These three PCR products were cloned into the *Xba*I site of pLP-1.2 using In-Fusion^®^ HD Cloning Kit (TaKaRa Bio) to give pLP-*phaA3*/*phaB2*/*phaC1*. All the overexpressed or disrupted genes used in this study are summarized in Table 2.

**Table 2:** Genes modified in this study

Gene name	KEGG ID	Annotated enzyme	Gene modification
*phaA1*	RSP_0745	Acetyl-CoA acetyltransferase	Over-expression
*phaA2*	RSP_1354	Acetyl-CoA acetyltransferase	Over-expression
*phaA3*	RSP_2197	Acetyl-CoA acetyltransferase	Over-expression
*phaA4*	RSP_3184	Acetyl-CoA acetyltransferase	Over-expression
*phaB1*	RSP_0747	Acetoacetyl-CoA reductase	Over-expression
*phaB2*	RSP_3963	Acetoacetyl-CoA reductase	Over-expression
*phaC1*	RSP_0382	Poly (3-hydroxyalkanoate) polymerase	Over-expression
*phaC2*	RSP_1257	Poly (3-hydroxyalkanoate) polymerase	Over-expression
*phaZ*	RSP_0383	Poly (3-hydroxyalkanoate) depolymerase	Disruption

### Construction of recombinant *R. sphaeroides* HJ strains

The pK18ms-*phaZ*/*spc*^*R*^ was introduced into *R. sphaeroides* HJ cells using conjugative DNA transfer via *E. coli* S17-1 [34]. Transformants raised by homologous recombination with pK18ms-*phaZ*/*spc*^*R*^ were selected using Km resistance and confirmed by PCR using the appropriate primer set. Transformants were aerobically grown in 5 ml of ASY liquid medium without antibiotics at 30 °C for 2 days. Grown cells were spread on ASY solid media containing Spc and sucrose, and aerobically grown at 30 °C for up to 3 days. The selected colony was confirmed as a *phaZ* disrupted strain (*R. sphaeroides* HJΔ*phaZ*) by PCR using the appropriate primer set.

The different derivatives of pLP-1.2 were introduced into *R. sphaeroides* HJ and HJΔ*phaZ* cells using conjugative DNA transfer via *E. coli* S17-1 [23]. Transformants harboring pLP-1.2 derivatives were selected using Km resistance. The *E. coli* and *R. sphaeroides* strains used in this study are summarized in Table 3.

**Table 3:** Strains and its relevant descriptions used in this study

Strain	Relevant description
*E. coli* NovaBlue	endA1, hsdR17, (rK- mK +), supE44, thi-1, gyrA96, relA1, lac, recA1/F’, [proAB + , lac Iq Z ΔM15, Tn10(tetr)]
*E. coli* S17-1	F-, thi, pro, hsdR, [RP4-2 Tc::Mu Km::Tn7 (Tp Sm)] (Sm^R^)
*R. sphaeroides* HJ	Wild type
*R. sphaeroides* HJΔ*phaZ*	Derivative strain of *R. sphaeroides* HJ; *phaZ*::s*pc* (Spc^R^)
*R. sphaeroides* HJ (pLP-1.2)	Derivative strain of *R. sphaeroides* HJ; pLP-1.2 (Km^R^)
*R. sphaeroides* HJΔ*phaZ* (pLP-1.2)	Derivative strain of *R. sphaeroides* HJ; *phaZ*::*spc* (Spc^R^) pLP-1.2 (Km^R^)
*R. sphaeroides* HJΔ*phaZ* (*phaA1*)	Derivative strain of *R. sphaeroides* HJ; *phaZ*::*spc* (Spc^R^) pLP-*phaA1* (Km^R^)
*R. sphaeroides* HJΔ*phaZ* (*phaA2*)	Derivative strain of *R. sphaeroides* HJ; *phaZ*::*spc* (Spc^R^) pLP-*phaA2* (Km^R^)
*R. sphaeroides* HJΔ*phaZ* (*phaA3*)	Derivative strain of *R. sphaeroides* HJ; *phaZ*::*spc* (Spc^R^) pLP-*phaA3* (Km^R^)
*R. sphaeroides* HJΔ*phaZ* (*phaA4*)	Derivative strain of *R. sphaeroides* HJ; *phaZ*::*spc* (Spc^R^) pLP-*phaA4* (Km^R^)
*R. sphaeroides* HJΔ*phaZ* (*phaB1*)	Derivative strain of *R. sphaeroides* HJ; *phaZ*::*spc* (Spc^R^) pLP-*phaB1* (Km^R^)
*R. sphaeroides* HJΔ*phaZ* (*phaB2*)	Derivative strain of *R. sphaeroides* HJ; *phaZ*::*spc* (Spc^R^) pLP-*phaB2* (Km^R^)
*R. sphaeroides* HJΔ*phaZ* (*phaC1*)	Derivative strain of *R. sphaeroides* HJ; *phaZ*::*spc* (Spc^R^) pLP-*phaC1* (Km^R^)
*R. sphaeroides* HJΔ*phaZ* (*phaC2*)	Derivative strain of *R. sphaeroides* HJ; *phaZ*::*spc* (Spc^R^) pLP-*phaC2* (Km^R^)
*R. sphaeroides* HJΔ*phaZ* (*phaA3*/*phaB2*/*phaC1*)	Derivative strain of *R. sphaeroides* HJ; *phaZ*::*spc* (Spc^R^) pLP-*phaA3*/*phaB2*/*phaC1* (Km^R^)

### PHB production

*R. sphaeroides* HJ derivative strains were aerobically grown on ASY solid media at 30 °C for 3 days. A single colony was inoculated into 5 ml of ASY liquid medium and aerobically grown at 30 °C for 3 days. Grown cells were inoculated into 20 ml of AAY liquid medium in 30 ml vials and incubated at 30 °C for 5 days. The vials, with a shell diameter of 33 mm were irradiated from the bottom with an 8.2 W/m^2^ of white light-emitting diode. The initial cell density (OD_660_) was adjusted to 0.25. The medium surface in the vial was covered with 10 ml of liquid paraffin to maintain anaerobic condition. More than three independent fermentation experiments were done for PHB production.

### PHB determination

PHB accumulated in *R. sphaeroides* cells was determined as previously reported [35]. Cell culture samples of 100 μl were pelleted by centrifugation (16,000×*g*, 10 min). The cell pellets were then rinsed twice with ultrapure water and resuspended in 2 ml of a 5% hypochlorous acid solution for digestion. Resuspended cells were digested by incubation at 40 °C for 2 h. Digested cell solutions were pelleted by centrifugation (16,000 × g, 10 min), and the supernatants were removed. The PHB pellets were rinsed twice with ultrapure water and dried by incubation at 90 °C for 30 min. Thereafter, 1.5 ml of concentrated sulfuric acid was added to dried PHB pellets and incubated at 90 °C for 30 min to degrade PHB to crotonic acid. The formation of crotonic acid was determined by measuring absorbance at a wavelength of 208 nm and calculated from the molar extinction coefficient of crotonic acid at 208 nm (14,100 M^−1^ cm^−1^) [35].

The DCW of *R. sphaeroides* strains was determined using 10 ml of residual cell cultures after 5 days of incubation. Cells were collected by centrifugation (16,000×*g*, 10 min) and rinsed twice with ultrapure water. Cell pellets were dried at 80 °C overnight. Dry cell pellets were weighted using an electronic balance.

### Enzyme assays

The activities of ACAT, AACR and PHAP in recombinant cells were determined by measuring the initial velocity of product formation. Recombinant *R. sphaeroides* strains grown for PHB production were sampled after 3 days of incubation; cells were collected by centrifugation (16,000×*g*, 1 min, 4 °C), and pellets were rinsed twice with ice-cold 20 mM potassium phosphate buffer. Cells were then resuspended in ice-cold 20 mM potassium phosphate buffer and disrupted by sonication. Disrupted cell mixtures were centrifuged (16,000×*g*, 10 min, 4 °C) to remove cellular debris, and the supernatants were used as crude extracts for enzyme assays.

To assay ACAT activity, we used a reaction mixture that contained 50 mM potassium phosphate buffer (pH 7.0), 0.1 mM acetyl-CoA, and 1 mM 5,5′-Dithiobis(2-nitrobenzoic acid) (DTNB). The reaction mixture (0.5 ml) was pre-incubated at 30 °C for 1 min in a quartz cell inserted into a V-670 spectrophotometer (JASCO, Tokyo, Japan) equipped with a MCB-100 thermostat (JASCO). After adding the enzyme and monitoring the absorbance of the solution at 412 nm, which arises from the formation of 5-thio-2-nitrobenzoic acid (TNB); the reaction was initiated at 30 °C. The concentration of TNB was determined using the molar extinction coefficient at 412 nm (13,600 M^−1^ cm^−1^).

To assay AACR activity, the reaction mixture contained 50 mM potassium phosphate buffer (pH 7.0), 0.1 mM acetoacetyl-CoA, and 0.1 mM NADPH. The reaction mixture was pre-incubated as described above. Addition of enzyme and monitoring the absorbance of the solution at 340 nm, which arises from NADPH, the reaction was initiated at 30 °C. The concentration of NADPH was determined using the molar extinction coefficient at 340 nm (62,200 M^−1^ cm^−1^).

PHAP activities were assayed as in the ACAT assay using a reaction mixture that contained 50 mM potassium phosphate buffer (pH 7.0), 0.1 mM (R)-3-hydroxybutanoyl-CoA, and 1 mM DTNB.

One unit was defined as the amount of enzyme that produces 1 μmol of the corresponding product per minute. Specific activities were determined from three independent experiments and are presented as the mean ± standard deviation. Protein concentrations were determined by the Bradford method.

### Analytical methods

Cell and crotonic acid concentrations were measured using a UVmini-1240 Spectrometer (Shimadzu, Kyoto, Japan). Residual acetate concentration in AAY medium during PHB production was determined by HPLC (Shimadzu) equipped with Shim-pack SCR-102H columns (Shimadzu). Operating condition were: 50 °C and a mobile phase flow rate of 0.75 ml min^−1^. The mobile phase was linearly graduated using 50% 5 mM *p*-toluenesulfonic acid and 50% 20 mM 2,2-Bis(hydroxymethyl)-2,2′,2″-nitrilotriethanol containing 5 mM *p*-toluenesulfonic acid, and 0.1 mM di-sodium dihydrogen ethylenediaminetetraacetate dihydrate. Detection was performed with a conductivity detector CDD-10A_VP_ (Shimadzu).

## Additional file

**Additional file 1: Table S1.** Volumetric PHB production, DCW, and PHB content of recombinant *R. sphaeroides* HJ strains. **Table S2**. Volumetric PHB production, DCW, and PHB content of recombinant *R. sphaeroides* HJ strains in various concentration of AS. **Table S3** PHB synthetic genes in *R. sphaeroides* 2.4.1 and its corresponding genes in *R. capsulatus* SB 1003 strain. **Table S4** Primers used in this study.

## Abbreviations

AACR: acetoacetyl coenzyme A reductase
ACAT: acetyl coenzyme A acetyltransferase
Acetoacetyl-CoA: acetoacetyl coenzyme A
Acetyl-CoA: acetyl coenzyme A
AS: ammonium sulfate
CoASH: coenzyme A
DCW: dry cell weight
DTNB: 5,5′-dithiobis(2-nitrobenzoic acid)
HPLC: high performance liquid chromatography
Km: kanamycin
Km^R^: kanamycin resistance
LB: Luria–Bertani
NADPH: nicotinamide adenine dinucleotide phosphate
PCR: polymerase chain reaction
PHA: poly (3-hydroxyalkanoates)
PHADP: poly (3-hydroxyalkanoate) depolymerase
PHAP: poly (3-hydroxyalkanoate) polymerase
PHB: poly (3-hydroxybutyrate)
PNS: purple non-sulfur
(R)-3-hydroxybutanoyl-CoA: (R)-3-hydroxybutanoyl coenzyme A
Sm: streptomycin
Sm^R^: streptomycin resistance
Spc: spectinomycin
Spc^R^: spectinomycin resistance
TNB: 5-thio-2-nitrobenzoic acid
