# Supplementary material for: Disruption of poly (3-hydroxyalkanoate) depolymerase gene and overexpression of three poly (3-hydroxybutyrate) biosynthetic genes improve poly (3-hydroxybutyrate) production from nitrogen rich medium by Rhodobacter sphaeroides
Source: Microb Cell Fact. 2019 Feb 26;18:40. doi: 10.1186/s12934-019-1088-y (PMC6390342; doi:10.1186/s12934-019-1088-y)
Supplement: Supplementary file 1 — Additional file 1: Table S1. Volumetric PHB production, DCW, and PHB content of recombinant R. sphaeroides HJ strains. Table S2. Volumetric PHB production, DCW, and PHB content of recombinant R. sphaeroides HJ strains in various concentration of AS. Table S3 PHB synthetic genes in R. sphaeroides 2.4.1 and its corresponding genes in R. capsulatus SB 1003 strain. Table S4 Primers used in this study. [file 12934_2019_1088_MOESM1_ESM.docx]

Additional file: Table S1-S4

Disruption of poly-3-hydroxyalkanoates depolymerase gene and over-expression of three poly-3-hydroxybutyrate synthetic genes improve poly-3-hydroxybutyrate production from nitrogen rich medium by Rhodobacter sphaeroides

Jyumpei Kobayashi,^1^ Akihiko Kondo^1,3,4^*

^1^ Graduate School of Science, Technology and Innovation, Kobe University, 1-1 Rokkodaicho, Nada-ku, Kobe, Hyogo 657-8501, Japan

^3^ Department of Chemical Science and Engineering, Graduate School of Engineering, Kobe University, 1-1 Rokkodaicho, Nada-ku, Kobe, Hyogo 657-8501, Japan

^4^ RIKEN Center for Sustainable Resource Science, 1-7-22 Suehiro-cho, Tsurumi-ku, Yokohama, Kanagawa 230-0045, Japan

*Corresponding author. Mailing address: Graduate School of Science, Technology and Innovation, Kobe University, 1-1 Rokkodaicho, Nada-ku, Kobe 657-8501, Japan. Phone and fax: 81-78-803-6196. E-mail: akondo@kobe-u.ac.jp

Table S1 Volumetric PHB production, volumetric PHB production rate, DCW, and PHB content of recombinant *R. sphaeroides* HJ strains

| Strain | Volmetric PHB producttion (g l^-1^) ^a^ | DCW (g l^-1^) ^a^ | PHB content (%)^a^ |
| --- | --- | --- | --- |
| HJ (pLP-1.2) | 0.38 ± 0.03 | 1.76 ± 0.04 | 21.8 ± 3.6 |
| HJΔ*phaZ* (pLP-1.2) | 1.10 ± 0.03 | 2.02 ± 0.01 | 53.6 ± 2.1 |
| HJΔ*phaZ* (*phaA1*) | 1.16 ± 0.01 | 2.06 ± 0.02 | 56.5 ± 0.2 |
| HJΔ*phaZ* (*phaA2)* | 1.13 ± 0.02 | 1.96 ± 0.04 | 57.8 ± 0.7 |
| HJΔ*phaZ* (*phaA3)* | 1.25 ± 0.01 | 2.18 ± 0.01 | 57.2 ± 0.9 |
| HJΔ*phaZ* (*phaA4)* | 1.22 ± 0.01 | 2.06 ± 0.06 | 59.4 ± 1.8 |
| HJΔ*phaZ* (*phaB1*) | 1.18 ± 0.01 | 2.08 ± 0.02 | 56.9 ± 0.4 |
| HJΔ*phaZ* (*phaB2)* | 1.20 ± 0.06 | 2.02 ± 0.02 | 59.6 ± 3.6 |
| HJΔ*phaZ* (*phaC1*) | 1.32 ± 0.02 | 2.12 ± 0.04 | 62.2 ± 1.9 |
| HJΔ*phaZ* (*phaC2)* | 1.29 ± 0.02 | 2.19 ± 0.09 | 56.3 ± 3.5 |

^a^, The data were measured and calculated after 5 days of culture.

Table S2 Volumetric PHB production, volumetric PHB production rate, DCW, and PHB content of recombinant *R. sphaeroides* HJ strains in various concentration of AS

| Strain | AS concentration (mM) | Volmetric PHB producttion (g l^-1^)^a^ | DCW (g l^-1^) ^a^ | PHB content (%)^a^ |
| --- | --- | --- | --- | --- |
| HJ (pLP-1.2) | - | 1.13 ± 0.09 | 2.37 ± 0.19 | 48.0 ± 2.9 |
| HJ (pLP-1.2) | 10 | 0.38 ± 0.03 | 1.76 ± 0.04 | 21.8 ± 2.1 |
| HJ (pLP-1.2) | 50 | 0.31 ± 0.04 | 1.91 ± 0.07 | 16.6 ± 2.7 |
| HJ (pLP-1.2) | 100 | 0.26 ± 0.03 | 2.05 ± 0.13 | 12.8 ± 2.0 |
| HJΔ*phaZ* (pLP-1.2) | - | 1.13 ± 0.05 | 2.18 ± 0.05 | 51.9 ± 2.5 |
| HJΔ*phaZ* (pLP-1.2) | 10 | 1.10 ± 0.03 | 2.02 ± 0.01 | 53.6 ± 2.1 |
| HJΔ*phaZ* (pLP-1.2) | 50 | 1.04 ± 0.05 | 1.95 ± 0.08 | 53.3 ± 2.7 |
| HJΔ*phaZ* (pLP-1.2) | 100 | 0.98 ± 0.02 | 1.84 ± 0.01 | 53.2 ± 0.6 |
| HJΔ*phaZ* (*phaA3*/*phaB2*/*phaC1*) | - | 1.88 ± 0.08 | 2.37 ± 0.08 | 79.8 ± 6.0 |
| HJΔ*phaZ* (*phaA3*/*phaB2*/*phaC1*) | 10 | 1.45 ± 0.03 | 2.16 ± 0.06 | 67.0 ± 1.9 |
| HJΔ*phaZ* (*phaA3*/*phaB2*/*phaC1*) | 50 | 1.20 ± 0.01 | 2.06 ± 0.03 | 58.4 ± 0.5 |
| HJΔ*phaZ* (*phaA3*/*phaB2*/*phaC1*) | 100 | 0.99 ± 0.05 | 1.92 ± 0.01 | 51.7 ± 2.7 |

^a^, The data were measured and calculated after 5 days of culture.

Table S3 PHB synthetic genes in *R. sphaeroides* 2.4.1 and its corresponding genes in *R. capsulatus* SB 1003 strain

| Gene | KEGG ID | | Nucleotide identity | Amino acid identity |
| --- | --- | --- | --- | --- |
|  | *R. sphaeroides* 2.4.1 | *R. capsulatus* SB 1003 |  |  |
| *phaA1* | RSP_0745 | RCAP_rcc03178 | 81.3 | 85.4 |
| *phaA2* | RSP_1354 | RCAP_rcc03449 | 74.7 | 72.4 |
| *phaA3* | RSP_2197 | RCAP_rcc00518 | 80.0 | 84.9 |
| *phaA4* | RSP_3184 | RCAP_rcc02992 | 72.3 | 69.6 |
| *phaB1* | RSP_0747 | RCAP_rcc03179 | 84.1 | 87.5 |
| *phaB2* | RSP_3963 | Not found | - | - |
| *phaC1* | RSP_0382 | RCAP_rcc00746 | 68.2 | 60.2 |
| *phaC2* | RSP_1257 | Not found | - | - |

Table S4 Primers used in this study

| Primer | Sequence (5’–3’) | Description |
| --- | --- | --- |
| phaA1F | ATCCACTAGTTCTAGATGACCAACGTGGTAATCGT | Construction of pLP-*phaA1* |
| phaA1R | TGGCGGCCGCTCTAGTCAGGGGCGCTCGAGGCACA | Construction of pLP-*phaA1* |
| phaA2F | ATCCACTAGTTCTAGATGTCCGACATCCTCGTCCT | Construction of pLP-*phaA2* |
| phaA2R | TGGCGGCCGCTCTAGTCAGACCCGCTCGAGCGCCA | Construction of pLP-*phaA2* |
| phaA3F | ATCCACTAGTTCTAGATGACGGAAGCCTATATCTA | Construction of pLP-*phaA3* and pLP-*phaA3*/*phaB2*/*phaC1* |
| phaA3R | TGGCGGCCGCTCTAGTCAGACGCGTTCGATGATGG | Construction of pLP-*phaA3* |
| phaA3R2 | GCTATCCTCCGGATCAGACGCGTTCGATGATGG | Construction of pLP-*phaA3*/*phaB2*/*phaC1* |
| phaA4F | ATCCACTAGTTCTAGATGGCAGCGGAACCGATCGT | Construction of pLP-*phaA4* |
| phaA4R | TGGCGGCCGCTCTAGTCAGATCACCTCCACCGCCA | Construction of pLP-*phaA4* |
| phaB1F | ATCCACTAGTTCTAGATGTCCAAGGTTGCTCTGGT | Construction of pLP-*phaB1* |
| phaB1R | TGGCGGCCGCTCTAGTCAGACGAAATACTGGCCGC | Construction of pLP-*phaB1* |
| phaB2F | ATCCACTAGTTCTAGATGACCAGGTTCGAAGGACG | Construction of pLP-*phaB2* |
| phaB2F2 | GATCCGGAGGATAGCATGACCAGGTTCGAAGGACG | Construction of pLP-*phaA3*/*phaB2*/*phaC1* |
| phaB2R | TGGCGGCCGCTCTAGTCAGTAGAAGATCCGGCCGC | Construction of pLP-*phaB2* |
| phaB2R2 | GCTATCCTCCGGATCAGTAGAAGATCCGGCCGCCG | Construction of pLP-*phaA3*/*phaB2*/*phaC1* |
| phaC1F | ATCCACTAGTTCTAGATGGCAACCGAAGAGCAGTC | Construction of pLP-*phaC1* |
| phaC1R | TGGCGGCCGCTCTAGTCAAGCCCCGCCCACCGCCG | Construction of pLP-*phaC1* |
| phaC2F | ATCCACTAGTTCTAGATGTCTGACATGAAGTGGAA | Construction of pLP-*phaC2* |
| phaC2F2 | GATCCGGAGGATAGCATGGCAACCGAAGAGCAGTC | Construction of pLP-*phaA3*/*phaB2*/*phaC1* |
| phaC2R | TGGCGGCCGCTCTAGTCAGCGTTGCAGGATGTAGG | Construction of pLP-*phaC2* and pLP-*phaA3*/*phaB2*/*phaC1* |
| phaZF | CGGGGATCCTCTAGAATGAAATATATGACAACC | Construction of pK18ms-*phaZ* |
| phaZR | GCCAAGCTTGCATGCTCTTGTTTGAGGCGGATCAG | Construction of pK18ms-*phaZ* |
| spcRF | GATCCCGACGCGGCCCCAGAACCTTGACCGAACGC | Construction of pK18ms-*phaZ*/*spc^R^* |
| spcRF | ACCTCGGTCGCGGCCTTATTTGCCGACTACCTTGG | Construction of pK18ms-*phaZ*/*spc^R^* |
